# Diverse Inhibitors of De Novo Purine Synthesis Promote AICAR‐Induced AMPK Activation and Glucose Uptake in L6 Myotubes

**DOI:** 10.1002/biof.70037

**Published:** 2025-08-12

**Authors:** Klemen Dolinar, Katarina Miš, Katja Šopar, Mateja Šutar, Meta Božič, Matic Kolar, Tim Hropot, Pablo M. Garcia‐Roves, Alexander V. Chibalin, Sergej Pirkmajer

**Affiliations:** ^1^ Institute of Pathophysiology, Faculty of Medicine University of Ljubljana Ljubljana Slovenia; ^2^ Department of Physiological Sciences, Faculty of Medicine and Health Sciences University of Barcelona Barcelona Spain; ^3^ Nutrition, Metabolism and Gene Therapy Group, Diabetes and Metabolism Program Institut D'investigació Biomédica de Bellvitge (IDIBELL) Barcelona Spain; ^4^ Department of Molecular Medicine and Surgery, Integrative Physiology Karolinska Institutet Stockholm Sweden; ^5^ National Research Tomsk State University Tomsk Russia

**Keywords:** AMP‐activated protein kinase (AMPK), folate metabolism, glucose uptake, insulin signaling, purine metabolism, skeletal muscle cells

## Abstract

Methotrexate, an immunosuppressant and anticancer drug, promotes glucose uptake and lipid oxidation in skeletal muscle via activation of AMP‐activated protein kinase (AMPK). Methotrexate promotes AMPK activation by inhibiting 5‐aminoimidazole‐4‐carboxamide ribonucleotide (ZMP) formyltransferase/inosine monophosphate (IMP) cyclohydrolase (ATIC), which converts ZMP, an endogenous purine precursor and an active form of the pharmacological AMPK activator AICAR, to IMP during de novo purine synthesis. In addition to methotrexate, inhibition of purine synthesis underpins the therapeutic effects of a number of commonly used immunosuppressive, anticancer, and antimicrobial drugs, raising the question of whether activation of AMPK in skeletal muscle could be a recurrent feature of these drugs. Using L6 myotubes, we found that AICAR‐induced AMPK activation and glucose uptake were enhanced by inhibitors of the conversion of IMP to GMP (mycophenolate mofetil) or of IMP to AMP (alanosine) as well as by indirect inhibitors of human (trimetrexate) and bacterial ATIC (sulfamethoxazole). 6‐Mercaptopurine, which inhibits the conversion of IMP to GMP and AMP, activated AMPK, increased glucose uptake, and suppressed insulin signaling, but did not enhance the effect of AICAR. As determined by measuring oxygen consumption rate, none of these agents suppressed mitochondrial function. Overall, our results indicate that IMP metabolism is a gateway for the modulation of AMPK and its metabolic effects in skeletal muscle cells.

## Graphical abstract

Mycophenolate mofetil, alanosine, trimetrexate, and sulfamethoxazole mimic methotrexate's effects by enhancing AICAR‐induced AMPK activation and glucose uptake in L6 myotubes. Collectively, our results suggest that IMP metabolism may serve as a gateway for the modulation of AMPK and its metabolic effects in skeletal muscle cells.

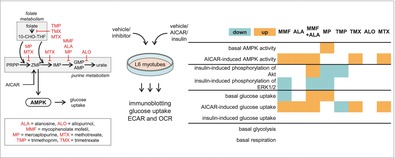

## 1 Introduction

Pharmacological activation of AMP‐activated protein kinase (AMPK)^1^ in skeletal muscle has emerged as a promising strategy for increasing glucose disposal, reducing insulin resistance, and alleviating hyperglycaemia in type 2 diabetes [1, 2]. Methotrexate, an immunosuppressant and antineoplastic drug [3, 4], promotes glucose uptake and lipid oxidation in skeletal muscle via activation of AMPK [5], alleviates glucose dysregulation in diabetic [6] and obese mice [7], and protects patients with rheumatoid and psoriatic arthritis against diabetes [8, 9]. Since methotrexate stimulates AMPK and its metabolic effects by inhibiting purine synthesis [5, 10, 11], we assumed that other inhibitors of purine metabolism, including commonly used immunosuppressant and antineoplastic drugs [12], might have a similar effect. By stimulating AMPK, inhibitors of purine metabolism, which are often used to treat inflammatory diseases and other conditions associated with glucose dysregulation [8, 12, 13, 14, 15, 16], could provide additional therapeutic benefit, especially over those immunosuppressants and antineoplastics that increase the risk of diabetes [17, 18].

**FIGURE 1:**
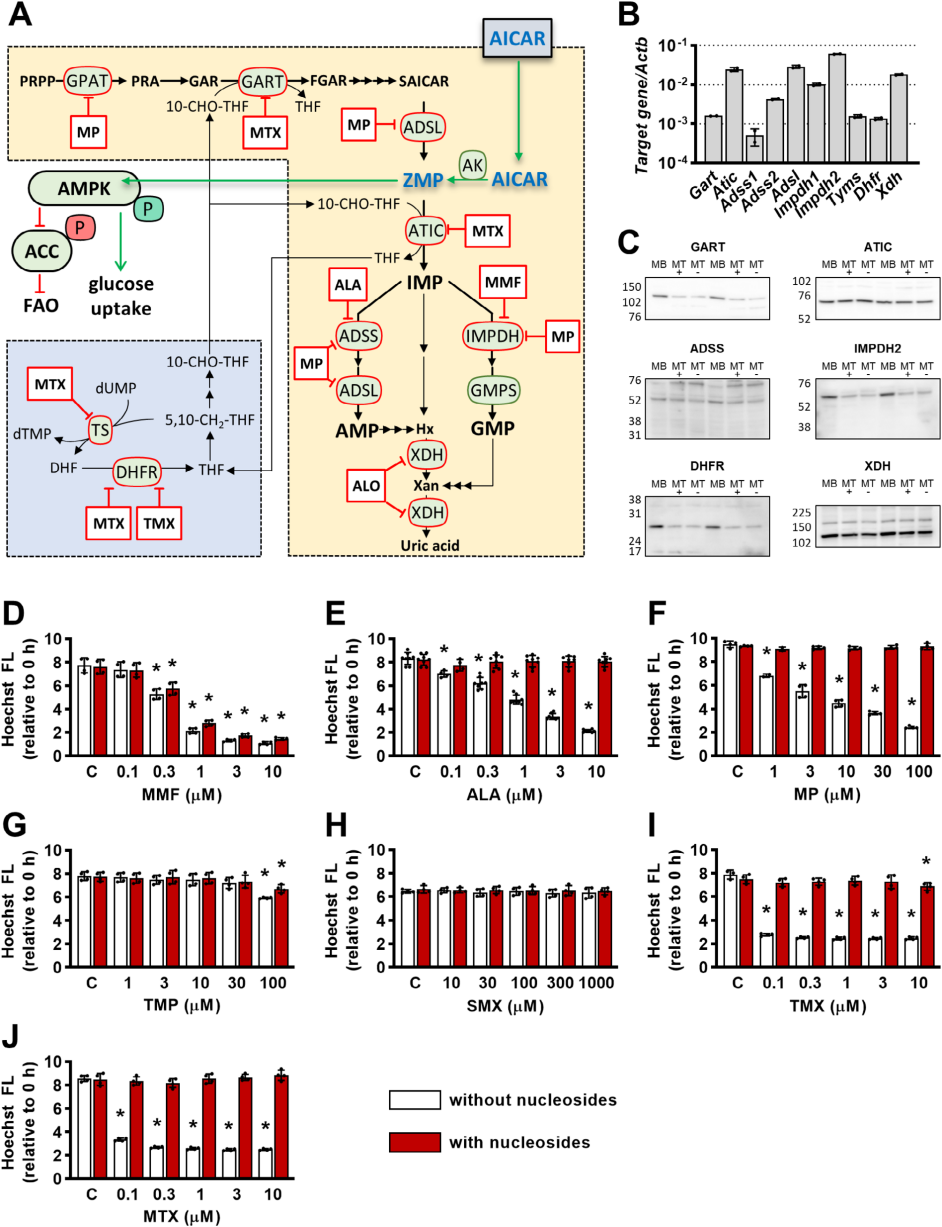
Assessment of sensitivity of L6 cells to inhibitors of purine metabolism. A: Purine metabolism and AMPK. The purine precursor ZMP is an AMPK activator. Methotrexate was shown to promote fatty acid oxidation (FAO) and glucose uptake via activation of AMPK in skeletal muscle tissue or cells [3, 19]. *Intermediates*: 5,10‐CH_2_‐THF, N^5^,N^10^‐methylene THF; 10‐CHO‐THF, N^10^‐Formyl‐THF; AMP, adenosine monophosphate; DHF, dihydrofolate; dUMP, deoxyuridine monophosphate; dTMP, deoxythymidine monophosphate; FGAR, formylglycinamide ribonucleotide; GAR, glycinamide ribonucleotide; GMP, guanosine monophosphate; Hx, hypoxanthine; IMP, inosine monophosphate; PRA, phosphoribosylamine; PRPP, 5‐phosphoribosyl‐1‐pyrophosphate; SAICAR, N‐succinyl‐5‐aminoimidazole‐4‐carboxamide ribonucleotide; THF, tetrahydrofolate; Xan, xanthine; ZMP, 5‐aminoimidazole‐4‐carboxamide ribonucleotide. *Enzymes*: ACC, acetyl‐coenzyme A carboxylase; ADSL, adenylosuccinate lyase; ADSS, adenylosuccinate synthetase; AMPK, AMP‐activated protein kinase; ATIC, 5‐aminoimidazole‐4‐carboxamide ribonucleotide formyltransferase/inosine monophosphate cyclohydrolase; DHFR, dihydrofolate reductase; GART, glycinamide ribonucleotide formyltransferase; GMPS, GMP synthetase; GPAT, glutamine phosphoribosylpyrophosphate amidotransferase; IMPDH, IMP dehydrogenase; TS, thymidylate synthetase; XDH, xanthine oxidase. *Inhibitors*: ALA, alanosine; ALO, allopurinol; FAO, fatty acid oxidation; MMF, mycophenolate mofetil; MP, mercaptopurine; MTX, methotrexate; TMP, trimethoprim; TMX, trimetrexate. “P” on AMPK and ACC indicates phosphorylation. (B, C) L6 Myotubes express enzymes of nucleotide and folate metabolism targeted by MTX, ALA, MMF, MP, TMX, and ALO. L6 cells were grown for 2 days in MEMα with nucleosides and 10% serum and then differentiated for 7 days in MEMα with nucleosides and 2% serum and for an additional day in MEMα without nucleosides and serum. Cells were then analyzed for expression of *Gart*, *Atic*, *Adss1*, *Adss2*, *Adsl*, *Impdh1*, *Impdh2*, *Tyms*, *Dhfr*, and *Xdh* genes (B). Expression of target genes was normalized to expression of Actin beta gene (*Actb*). Graphs show means with SD (*n* = 2). *Tyms*: Thymidylate synthetase. In addition, cells were analyzed for protein expression of GART, ATIC, ADSS, IMPDH2, DHFR, and XDH on day 2 (myoblasts; MB), day 9 (myotubes after 7 days in MEMα with nucleosides and 2% serum; MT+) and day 10 (myotubes after 7 days in MEMα with nucleosides and 2% serum and 1 day in MEMα without nucleosides and serum; MT‐) of culture (C). Numbers next to blots indicate position and molecular weight (in kDa) of molecular weight markers. (D–J) Effect of MMF, ALA, MP, TMP, sulfamethoxazole (SMX), TMX and MTX on proliferation of L6 myoblasts in absence or presence of nucleosides. L6 myoblasts were grown in absence of nucleosides for 24 h and then treated with MMF (0.1–10 μM) (D), ALA (0.1–10 μM) (E), MP (1–100 μM) (F), TMP (1–100 μM) (G), SMX (10–1000 μM) (H), TMX (0.1–10 μM) (I), MTX (0.1–10 μM) (J) or vehicle (control, C) in absence or in presence of nucleosides for 48 h. Cell cultures before and after the treatment were analyzed for DNA content with Hoechst assay. Hoechst fluorescence (Hoechst FL) after the treatment was expressed relative to Hoechst fluorescence before the treatment (0 h). Graphs show means with SD (*n* = 4–8). *p < 0.05 versus respective (without or with nucleosides) control, two‐way ANOVA with Dunnett's test.

In skeletal muscle, methotrexate promotes AMPK activation, glucose uptake, and lipid oxidation induced by 5‐aminoimidazole‐4‐carboxamide ribonucleotide (ZMP) [5] (Figure 1A). ZMP is both an endogenous precursor of inosine monophosphate (IMP) in the de novo purine synthesis pathway [20, 21, 22, 23] and the active (phosphorylated) form of a widely used experimental AMPK activator, 5‐aminoimidazole‐4‐carboxamide ribofuranoside (AICAR) [24, 25, 26] (Figure 1A). As an AMP analogue, ZMP binds to AMPK and activates it directly [24, 26, 27], but its concentrations in skeletal muscle are physiologically low and may remain below the threshold for AMPK activation even in the presence of AICAR [5, 28, 29]. Methotrexate increases ZMP concentrations and facilitates AMPK activation by inhibiting 5‐aminoimidazole‐4‐carboxamide ribonucleotide formyltransferase/inosine monophosphate cyclohydrolase (ATIC) [5, 10, 30, 31], an enzyme that catalyzes the conversion of ZMP to IMP in the de novo purine synthesis pathway [22, 23].

Once formed, IMP is used for the de novo synthesis of GMP or AMP (Figure 1A). Mycophenolate mofetil, an immunosuppressant, and 6‐mercaptopurine, an antineoplastic and immunosuppressant drug, inhibit IMP dehydrogenase (IMPDH) [19, 32, 33, 34], which catalyzes the first, rate‐limiting step, in the synthesis of GMP from IMP. 6‐Mercaptopurine also inhibits adenylosuccinate synthetase (ADSS) and adenylosuccinate lyase (ADSL) [32], which catalyze the synthesis of AMP from IMP. Inhibition of IMPDH by mycophenolic acid (the active form of mycophenolate mofetil) increased ZMP levels in cancer cells [35], suggesting that inhibitors of GMP and/or AMP synthesis suppress ZMP clearance and facilitate AMPK activation, mimicking the inhibition of ATIC by methotrexate [31].

Methotrexate suppresses ATIC both directly [36, 37] and through inhibition of dihydrofolate reductase (DHFR) [38] (Figure 1A). Inhibition of DHFR by methotrexate or trimetrexate, an antimicrobial drug, leads to a decrease in 10‐formyl‐tetrahydrofolates, which are required for the conversion of ZMP to IMP, while inhibitory dihydrofolates increase [30], resulting in suppression of ATIC and accumulation of ZMP [30]. In bacteria, sulfonamides and trimethoprim (inhibitor of bacterial DHFR), typically used in combination as trimethoprim‐sulfamethoxazole to treat various infections [39], inhibit two consecutive steps in the synthesis of tetrahydrofolate, which also results in ZMP accumulation [40], underscoring that inhibitors of folate synthesis act as indirect ATIC inhibitors.

In the present study, we asked whether inhibitors of GMP (mycophenolate mofetil) [19, 34], AMP (alanosine) [41], or GMP and AMP synthesis (6‐mercaptopurine) [32, 33] and indirect inhibitors of human (trimetrexate) [30] or bacterial ATIC (sulfamethoxazole and trimethoprim) [40] mimic effects of methotrexate and promote AMPK activation and glucose uptake in cultured myotubes. Their effects on insulin signaling and mitochondrial respiration were also assessed.

## 2 Experimental Procedures

### 2.1 Materials

Cell culture flasks and plates were from Sarstedt and Techno Plastic Products, respectively. Minimum Essential Medium α (MEMα), Fetal Bovine Serum (FBS), Pen Strep (penicillin and streptomycin), Amphotericin B, High‐Capacity cDNA Reverse Transcription Kit, TaqMan Universal PCR Master Mix II, TaqMan gene expression assays, Pierce Bicinchoninic Acid (BCA) Protein Assay Kit, Pierce 660 nm Protein Assay Reagent, Ionic Detergent Compatibility Reagent, and Pierce Enhanced Chemiluminescence (ECL) Western Blotting Substrate were from Thermo Fisher Scientific. Nucleosides for MEMα (500×) were from Sartorius/Biological Industries. E.Z.N.A. HP Total RNA Kit was from Omega Bio‐tek. 4%–12% Criterion XT Bis‐Tris polyacrylamide protein gels and XT MES electrophoresis buffer were from Bio‐Rad. Amersham ECL Full‐Range Rainbow Molecular Weight Markers were from Cytiva. Polyvinylidene fluoride (PVDF) membrane and Immobilon Crescendo Western Horseradish Peroxidase (HRP) substrate were from Merck. Dry skimmed milk was from Pomurske mlekarne. 2‐[1,2‐^3^H]‐deoxy‐glucose (1 mCi/mL) and liquid scintillation cocktail Aquasol 2 were from PerkinElmer. Hoechst 33342 was from Merck/Sigma‐Aldrich. Compounds used in experiments: mycophenolate mofetil (Merck/Sigma‐Aldrich #SML0284), alanosine (Cayman Chemical #19545), 6‐mercaptopurine (Merck/Sigma‐Aldrich #852678), trimethoprim (Cayman Chemical #16473), sulfamethoxazole (Cayman Chemical #23613), trimetrexate (CI‐898) (Cayman Chemical #26389), allopurinol (Cayman Chemical #10012597), methotrexate (Merck/Calbiochem #454126), 5‐aminoimidazole‐4‐carboxamide 1‐β‐D‐ribofuranoside (AICAR) (Cayman Chemical #10010241), human insulin Actrapid (Novo Nordisk), oligomycin (Merck/Calbiochem #495455), carbonyl cyanide 4‐(trifluoromethoxy)phenylhydrazone (FCCP) (Merck/Sigma‐Aldrich #C2920), rotenone (Merck/Calbiochem #557368), antimycin A (Merck/Sigma‐Aldrich #A8674). All other reagents, unless specified otherwise, were from Merck/Sigma‐Aldrich or VWR.

### 2.2 Cell Culture

Rat skeletal muscle cell line L6 was from American Type Culture Collection (ATCC, #CRL‐1458). L6 myoblasts were cultured in MEMα with 1× nucleosides (adenosine, cytidine, guanosine, uridine, 2′‐deoxyadenosine, 2′‐deoxycytidine HCl, 2′‐deoxyguanosine and 2′‐deoxythymidine at 10 mg/L), 10% (v/v) FBS, Pen Strep (50 units/mL penicillin and 50 μg/mL streptomycin) and Amphotericin B (0.75 μg/mL) at 37°C in humidified air with 5% (v/v) CO_2_. To differentiate myoblasts into myotubes, myoblasts were grown in the presence of 10% (v/v) FBS until ~80% confluent and then for an additional 6–8 days in the presence of 2% (v/v) FBS.

### 2.3 Quantification of Total Proteins

When cells were lysed with sodium dodecyl sulfate (SDS), total proteins were quantified with BCA protein assay using BCA protein assay kit. When cells were lysed with Laemmli sample buffer (62.5 mM tris (pH 6.8, adjusted with HCl), 2% (w/v) SDS, 10% (w/v) glycerol, 5% (v/v) 2‐mercaptoethanol, 0.002% (w/v) bromophenol blue), total proteins were quantified with 660 nm protein assay using 660 nm protein assay reagent supplemented with ionic detergent compatibility reagent. Assays were performed according to the manufacturer's instructions. Bovine serum albumin (BSA) (62.5–1000 μg/mL in water) was used as the protein standard. Absorbance was measured with Epoch microplate spectrophotometer (Agilent/BioTek).

### 2.4 Analysis of Gene Expression With Quantitative Real‐Time Polymerase Chain Reaction

RNA was extracted with E.Z.N.A. HP Total RNA Kit and reverse transcribed to cDNA with High‐Capacity cDNA Reverse Transcription Kit. Quantitative real‐time polymerase chain reaction (qPCR) was performed with QuantStudio 3 Real‐Time PCR System (Thermo Fisher Scientific) using TaqMan Universal PCR Master Mix II and TaqMan gene expression assays for *Gart* (Rn01477298_m1), *Atic* (Rn00578818_m1), *Adss1* (Rn01430183_m1), *Adss2* (Rn02103847_s1), *Adsl* (Rn01768239_m1), *Impdh1* (Rn01455843_g1), *Impdh2* (Rn01640111_g1), *Tyms* (Rn01418709_m1), *Dhfr* (Rn04342282_g1), *Xdh* (Rn00567654_m1) and *Actb* (4352931). Expression of target genes is reported as the gene expression ratio:

$$\frac{\text{target gene mRNA}}{\text{Actb mRNA}}=\frac{{\left(1+E_{\text{Actb}}\right)}^{{\mathrm{Cq}}_{\text{Actb}}}}{{\left(1+E_{\text{target}}\right)}^{{\mathrm{Cq}}_{\text{target}}}}$$

where Cq is quantification cycle and E is average amplification efficiency of an assay expressed as a value between 0 (no amplification) and 1 (100% amplification efficiency). The amplification efficiency was determined with LinRegPCR software [42].

### 2.5 Analysis of Cell Proliferation With Hoechst 33342 Assay

Cell proliferation was assessed from changes in total cell culture DNA content, which was determined with fluorescent DNA dye Hoechst 33342 as described [11]. L6 cells were seeded in 24‐well cell culture plates at a cell density of 1 × 10^4^ cells/well in growth medium without nucleosides. 24 h later, cell culture medium was replaced with fresh growth medium without or with nucleosides (adenosine, cytidine, guanosine, uridine, 2′‐deoxyadenosine, 2′‐deoxycytidine HCl, 2′‐deoxyguanosine and 2′‐deoxythymidine at 10 mg/L) and without or with inhibitors of de novo purine synthesis. Cell cultures before and after treatment were washed with phosphate‐buffered saline (PBS: 137 mM NaCl, 2.7 mM KCl, 10 mM Na_2_HPO_4_, 1.8 mM KH_2_PO_4_, pH 7.4) and stored at‐20°C until analysis. For analysis, cell cultures were lysed with 0.04% (w/v) SDS in water. Lysates were transferred to a 96‐well plate and mixed with an equal volume of tris‐NaCl buffer (50 mM tris, 100 mM NaCl, pH 8.3 (adjusted with HCl)) with 10 μg/mL Hoechst 33342. Samples were incubated for 15 min at room temperature, and then Hoechst fluorescence was measured with a VICTOR3 microplate reader (PerkinElmer) using a 355 nm excitation filter and a 460 nm emission filter.

### 2.6 Analysis of Protein Expression and Phosphorylation With Immunoblotting

Immunoblotting was performed as described [11]. After the treatment, cells were washed with cold PBS, lysed in Laemmli sample buffer, sonicated, and heated at 60°C for 20 min. Proteins were separated by their molecular weight with electrophoresis in 4%–12% polyacrylamide protein gels in electrophoresis buffer and transferred to PVDF membrane with wet electrotransfer in transfer buffer (31.3 mM tris base, 240 mM glycine, 10% (v/v) methanol, 0.01% (w/v) SDS). After the transfer, membranes were stained with Ponceau S (0.1% (w/v) Ponceau S in 5% (v/v) acetic acid) to evaluate the uniformity of protein loading and transfer. Membranes were then destained in tris‐buffered saline with Tween 20 (TBST: 20 mM tris (pH 7.5, adjusted with HCl), 150 mM NaCl, 0.02% (v/v) Tween 20) and blocked with 5% (w/v) dry skimmed milk in TBST for 1 h at room temperature. Following the blocking, membranes were incubated with primary antibodies (Table 1) in primary antibody buffer (20 mM tris (pH 7.5, adjusted with HCl), 150 mM NaCl, 0.1% (w/v) BSA and 0.1% (w/v) sodium azide) overnight at 4°C and then with goat anti‐rabbit IgG‐HRP conjugate (BioRad #1706515), diluted 1:10,000–1:20,000 in TBST with 5% (w/v) dry skimmed milk for 1 h at room temperature. Finally, membranes were incubated with ECL substrate and then the signal was captured on x‐ray films (CP‐BU NEW x‐ray films, Agfa HealthCare) or with FUSION FX6 (Vilber). Films were processed with Curix 60 film processor (Agfa HealthCare) and scanned with GS‐800 Densitometer (Bio‐Rad). Bands were analyzed with Quantity One 1‐D Analysis Software (Bio‐Rad). Membranes were first probed for phosphosites, then stripped of antibodies in stripping buffer (62.5 mM tris (pH 6.8, adjusted with HCl), 2% (w/v) SDS, 0.7% (v/v) 2‐mercaptoethanol), re‐blocked, and probed for the corresponding total proteins as described above. Before incubation with primary antibodies against the corresponding total proteins, membranes were incubated with secondary antibody and ECL substrate and examined for signal as described above. The absence of signal confirmed that membranes were successfully stripped of primary antibodies against phosphoproteins.

**TABLE 1:** List of antibodies used for immunoblotting.

Target	Primary antibody			
	Company and product number	Host organism	Clonality	Dilution
pACC Ser79	CST #3661	Rabbit	pAb	1:1000
ACC	CST #3676	Rabbit	mAb	1:1000
pAMPKα Thr172	CST #2535	Rabbit	mAb	1:1000
AMPKα	CST #2532	Rabbit	pAb	1:1000
pAkt Ser473	CST #4060	Rabbit	mAb	1:2000
Akt	CST #4691	Rabbit	mAb	1:1000
pERK1/2 Thr202/Tyr204	CST #4370	Rabbit	mAb	1:20,000
ERK1/2	CST #4695	Rabbit	mAb	1:1000
GART	Proteintech #67939–1‐Ig	Mouse	mAb	1:10,000
ADSS	Proteintech #16373–1‐AP	Rabbit	pAb	1:1000
XDH	Proteintech #55156–1‐AP	Rabbit	pAb	1:1000
IMPDH2	Proteintech #12948–1‐AP	Rabbit	pAb	1:10,000
ATIC	antibodies‐online #ABIN7005053	Rabbit	pAb	1:1000
DHFR	Proteintech #15194–1‐AP	Rabbit	pAb	1:2000

Abbreviations: CST, Cell Signaling Technology; mAb, monoclonal antibody; p, phospho; pAb, polyclonal antibody.

### 2.7 Analysis of Glucose Uptake With 2‐Deoxy‐Glucose Uptake Assay

Glucose uptake was determined by measuring the uptake of tritium (^3^H)‐labeled 2‐deoxy‐glucose (2DG) as described [11]. Cells were washed with HEPES‐buffered saline (HBS: 140 mM NaCl, 20 mM HEPES, 5 mM KCl, 2.5 mM MgCl_2_, 1 mM CaCl_2_, pH 7.4 (adjusted with NaOH)), incubated in HBS with 10 μM 2DG (unlabelled) and 1 μCi/mL 2‐[1,2‐^3^H]‐DG for 10 min at 37°C, washed with cold PBS with 25 mM glucose, and lysed with 0.04% (w/v) SDS in water. Cell lysates were then analyzed for protein content with BCA protein assay or mixed with liquid scintillation cocktail and analyzed for radioactivity with MicroBeta TriLux scintillation counter (PerkinElmer). The amount of 2DG in samples was determined from the radioactivity of samples using a standard (known amount of 2‐[1,2‐^3^H]‐DG) and is expressed in pmol of 2DG/min/mg of proteins.

### 2.8 Analysis of Mitochondrial Respiration Rate and Glycolysis Rate With Seahorse XF Analyzer

Mitochondrial respiration rate and glycolysis rate were calculated from oxygen consumption rate (OCR) and extracellular acidification rate (ECAR) measured with Seahorse XF Analyzer (Agilent). L6 cells were seeded in Seahorse XF24 cell culture microplates (Agilent) and differentiated into myotubes. Myotubes were washed with PBS and incubated in MEMα for 23 h, with the first 8 h without and the next 15 h with vehicle or inhibitors of purine metabolism. Cell culture medium was then replaced with assay medium (Seahorse XF DMEM supplemented with 10 mM glucose, 2 mM glutamine and 1 mM pyruvate (all from Agilent)) with vehicle or inhibitors of purine metabolism. Cells were incubated for additional 60 min and then analyzed for OCR and ECAR before (basal OCR and basal ECAR) and after treatment with 1 μM oligomycin, 2 μM FCCP, and 0.75 μM rotenone + 0.75 μM antimycin A (R + AA). OCR (in pmol O_2_/min) and ECAR (in mpH/min) were normalized to total protein content (in μg), which was determined with BCA protein assay after lysis of cells with 0.1% (w/v) SDS in water. Normalized OCR and ECAR were used to calculate basal respiration (*basal OCR* − *OCR after R + AA*), proton leak‐linked respiration (*OCR after OM* − *OCR after R + AA*), ATP production‐linked respiration (*basal OCR* − *OCR after OM*), maximal respiration (*OCR after FCCP* − *OCR after R + AA*), spare respiratory capacity (*maximal respiration* − *basal respiration*), total proton efflux rate (PER; expressed in pmol H^+^/min/μg protein; calculated by Seahorse Analyzer), mitochondrial PER (*0.60* × *[OCR* − *OCR after R + AA]*) and glycolytic PER (*total PER* − *mitochondrial PER*).

### 2.9 Statistical Analysis

Data are presented as means with standard deviation (SD). Statistical analysis was performed with GraphPad Prism 8 (GraphPad Software) using ANOVA with Dunnett's or Bonferroni's test. The difference between two groups was considered statistically significant when *p* was < 0.05.

## 3 Results

### 3.1 Assessment of Sensitivity of L6 Cells to Inhibitors of Purine Metabolism

As assessed by PCR (Figure 1B), L6 myotubes expressed *Impdh*, *Adss*, *Adsl*, and *Dhfr*, which encode enzymes that are inhibited by mycophenolate mofetil (IMPDH), alanosine (experimental inhibitor of ADSS), 6‐mercaptopurine (IMPDH, ADSS, and ADSL), trimetrexate (DHFR), and/or methotrexate (ATIC, DHFR) (Figure 1A,B). *Gart*, *Tyms*, and *Xdh*, encoding glycinamide ribonucleotide transformylase (GART) and thymidylate synthase (TYMS), which are both inhibited by methotrexate, and xanthine dehydrogenase (XDH), a key purine‐degrading enzyme, were also expressed (Figure 1A,B). Expression of GART, ATIC, ADSS, IMPDH, DHFR, and XDH was verified also at the protein level (Figure 1C).

To determine whether L6 cells are sensitive to inhibition of de novo purine synthesis, proliferating L6 myoblasts were treated with the selected inhibitors for 48 h (Figure 1D–J). After a 48‐h treatment in the nucleoside‐free medium, the proliferation of L6 myoblasts was inhibited by mycophenolate mofetil (half‐maximal effective concentration (EC50) ≈0.4 μM) (Figure 1D), alanosine (EC50 ≈1.4 μM) (Figure 1E), 6‐mercaptopurine (EC50 ≈3.0 μM) (Figure 1F), trimetrexate (EC50 < 0.1 μM) (Figure 1I), and methotrexate (EC50 < 0.1 μM) (Figure 1J). The addition of nucleosides to cell medium, which obviated the need for de novo purine synthesis, abolished the antiproliferative effects of these drugs except for mycophenolate mofetil. Sulfamethoxazole, an inhibitor of bacterial dihydropteroate synthase (not present in humans), had no effect on L6 myoblasts (Figure 1H), while trimethoprim, an inhibitor of bacterial DHFR and a very weak inhibitor of human DHFR [43], reduced their proliferation slightly (EC50 > 100 μM) (Figure 1G).

### 3.2 Mycophenolate Mofetil, Alanosine, Trimetrexate, and Sulfamethoxazole Promote AICAR‐Induced AMPK Activation and Glucose Uptake in L6 Myotubes

**FIGURE 2:**
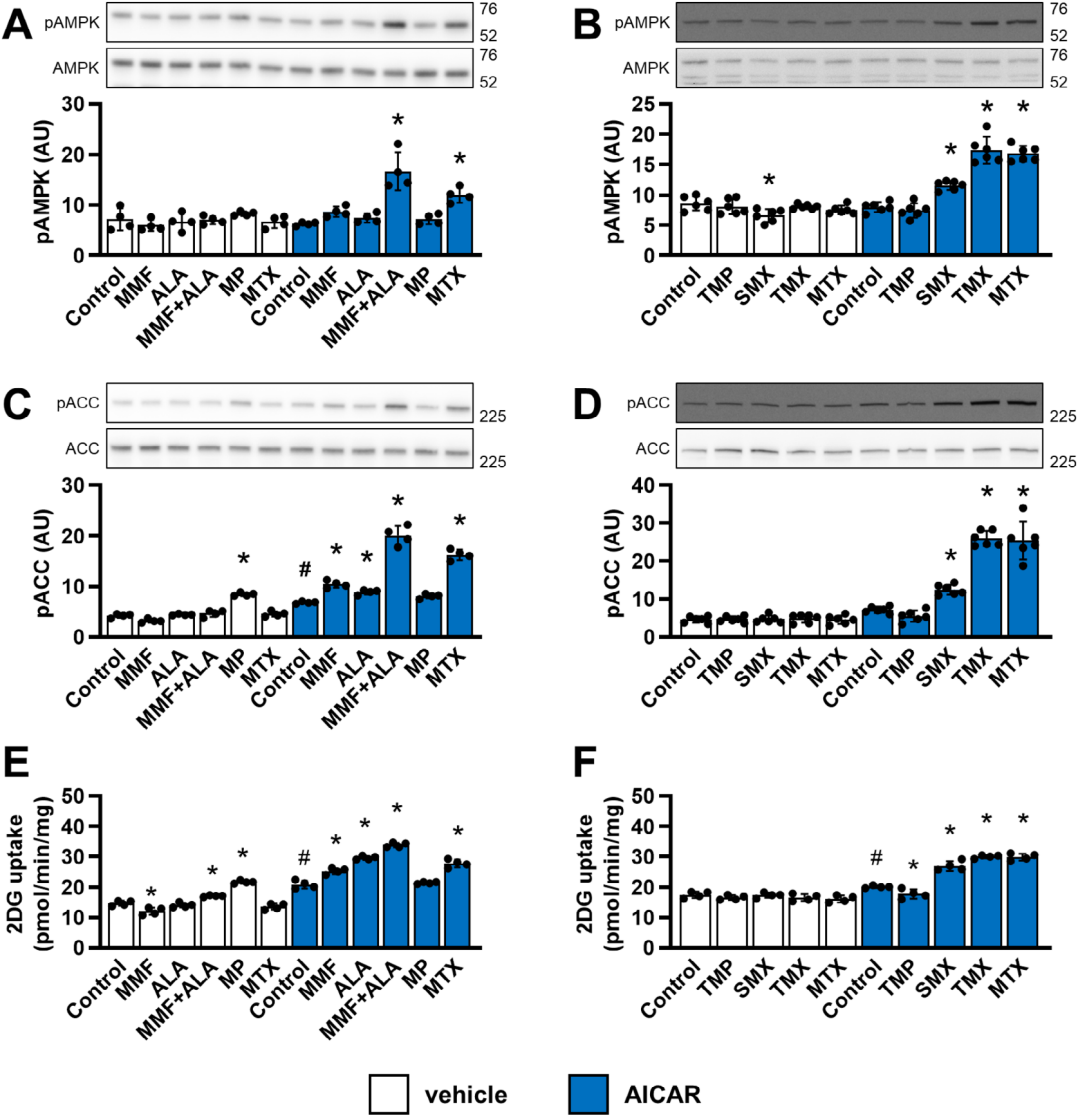
Mycophenolate mofetil, alanosine, trimetrexate, and sulfamethoxazole promote AICAR‐induced AMPK activation and glucose uptake in L6 myotubes. L6 myotubes were incubated in nucleoside‐ and serum‐free MEMα for 24 h and treated with 5 μM mycophenolate mofetil (MMF), 5 μM alanosine (ALA), MMF + ALA, 50 μM mercaptopurine (MP), 20 μM trimethoprim (TMP), 500 μM sulfamethoxazole (SMX), 5 μM trimetrexate (TMX), 5 μM methotrexate (MTX) or vehicle (Control) for the last 16 h of these 24 h and with 1 mM AICAR or vehicle for the last 60 min of these 24 h for analysis of protein phosphorylation or with 2 mM AICAR or vehicle for the last 5 h of these 24 h for analysis of glucose uptake. Following the treatment, cells were analyzed for phospho AMPKα Thr172 (pAMPK) and AMPKα (AMPK) (A, B) and phospho ACC Ser79 (pACC) and ACC (C, D) with immunoblotting or for glucose uptake with 2‐deoxy‐glucose (2DG) uptake assay (E, F). 2DG uptake was expressed in pmol of 2DG/min/mg of total proteins. Graphs show means with SD (*n* = 4–6). Images show representative blots. Numbers next to blots indicate molecular weight (in kDa) of the first marker below and/or above the bands on the blots. *p < 0.05 versus respective (vehicle or AICAR) control, #*p* < 0.05 AICAR control versus vehicle control; one‐way ANOVA with Bonferroni's test. AU, arbitrary units.

Methotrexate inhibits the conversion of ZMP to IMP and enhances AICAR‐induced AMPK activation and glucose uptake in cultured myotubes [5, 11]. To test whether inhibition of the IMP conversion to GMP and/or AMP has a similar effect, L6 myotubes were treated with the selected inhibitors in the presence or absence of AICAR (Figure 2A,C,E). Activation of AMPK was estimated by measuring phosphorylation of the catalytic AMPK α‐subunit at Thr172 (Figure 2A,B) and phosphorylation of its substrate acetyl‐coenzyme A carboxylase (ACC) at Ser79 (Figure 2C,D).

In the presence of AICAR, methotrexate and co‐treatment with mycophenolate mofetil and alanosine increased the phosphorylation of AMPK (Figure 2A) and ACC (Figure 2C), which was paralleled by an increase in glucose uptake (Figure 2E). When used singly, mycophenolate mofetil and alanosine did not alter the phosphorylation of AMPK in AICAR‐treated myotubes(Figure 2), although they increased AICAR‐induced phosphorylation of ACC (Figure 2C) and glucose uptake (Figure 2E). 6‐Mercaptopurine increased the phosphorylation of ACC (Figure 2C) and glucose uptake (Figure 2E) in the absence of AICAR, but had no significant effect when AICAR was present. These results suggested that mycophenolate mofetil and alanosine, but not 6‐mercaptopurine, mimic the effects of methotrexate on AICAR‐induced AMPK activation and glucose uptake.

Inhibition of DHFR disrupts regeneration of tetrahydrofolate and thereby suppresses ATIC and purine synthesis [30, 40]. L6 myotubes were treated with trimetrexate (inhibitor of human DHFR), trimethoprim (inhibitor of bacterial DHFR and a very weak inhibitor of human DHFR [43]), sulfamethoxazole (inhibitor of bacterial dihydrofolate synthesis), and methotrexate (Figure 2B,D,F). In the presence of AICAR, trimetrexate, sulfamethoxazole, and methotrexate increased AMPK and ACC phosphorylation (Figure 2B,D) and glucose uptake (Figure 2F). Trimethoprim did not alter AMPK and ACC phosphorylation (Figure 2B,D) and even somewhat reduced glucose uptake (Figure 2F) in AICAR‐treated myotubes. These results indicated that trimetrexate and sulfamethoxazole enhance AICAR‐induced AMPK activation and glucose uptake.

### 3.3 Effect of Inhibitors of de Novo Purine Synthesis on Mitochondrial Respiration and Glycolysis in L6 Myotubes

**FIGURE 3:**
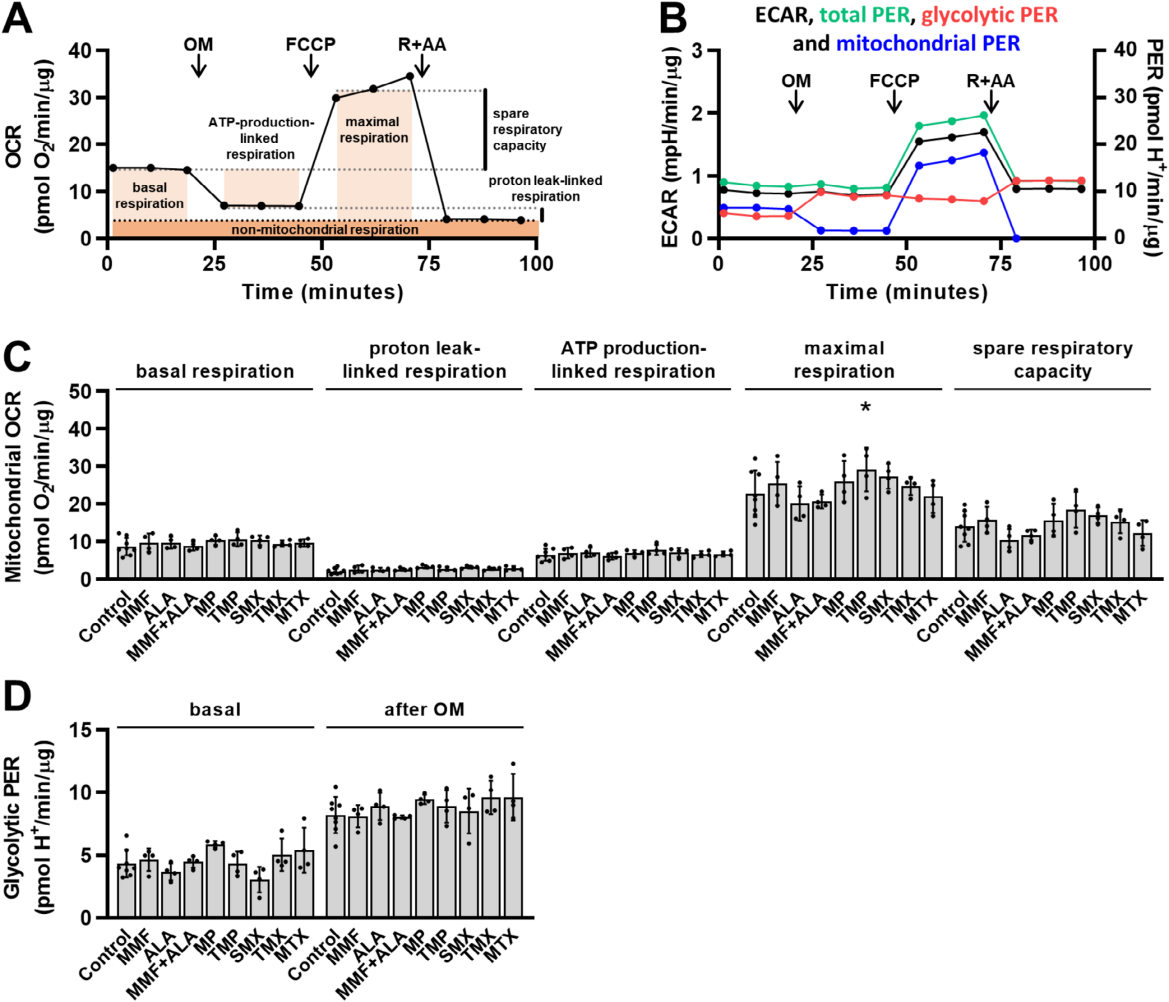
Effect of inhibitors of de novo purine synthesis on mitochondrial respiration and glycolysis in L6 myotubes. L6 myotubes were incubated in nucleoside‐ and serum‐free MEMα for 23 h and treated with 5 μM mycophenolate mofetil (MMF), 5 μM alanosine (ALA), MMF + ALA, 50 μM mercaptopurine (MP), 20 μM trimethoprim (TMP), 500 μM sulfamethoxazole (SMX), 5 μM trimetrexate (TMX), 5 μM methotrexate (MTX) or vehicle (Control) for the last 15 h of these 23 h. Cell culture medium was then replaced with Seahorse assay medium with MMF, ALA, MMF + ALA, MP, TMP, SMX, TMX, MTX, or vehicle. Cells were incubated for additional 60 min and then analyzed for oxygen consumption rate (OCR) and extracellular acidification rate (ECAR) before and after treatment with 1 μM oligomycin (OM), 2 μM FCCP, and 0.75 μM rotenone + 0.75 μM antimycin A (R + AA) using Seahorse Analyzer. OCR (in pmol O_2_/min) and ECAR (in mpH/min) were normalized to total protein content (in μg). Basal respiration, proton leak‐linked respiration, ATP production‐linked respiration, maximal respiration, spare respiratory capacity, and total, mitochondrial, and glycolytic proton efflux rate (PER; expressed in pmol H^+^/min/μg protein) were calculated. (A) and (B) show OCR curve (A) and ECAR, total PER, mitochondrial PER, and glycolytic PER curves (B) of control cells (mean of four control cultures from one of two independent experiments). (C) shows means with SD of basal respiration, proton leak‐linked respiration, ATP production‐linked respiration, maximal respiration, and spare respiratory capacity (*n* = 4–8). (D) shows means with SD of glycolytic PER before (basal) and after the treatment with OM (*n* = 4–8). *p < 0.05 versus respective control, two‐way ANOVA with Dunnett's test.

Since many compounds that act as AMPK activators do so by suppressing mitochondrial function [26], the effect of inhibitors of purine synthesis on the oxygen consumption rate (OCR) and extracellular acidification rate (ECAR) in L6 myotubes was assessed. As shown in Figure 3A,B, OCR and ECAR were measured before and after inhibition of ATP synthase with oligomycin, after uncoupling of oxidative phosphorylation with FCCP, and after inhibition of complexes I and III of the electron transport system with rotenone and antimycin A. Treatment with inhibitors of purine synthesis did not significantly alter basal respiration, proton leak‐linked respiration, ATP production‐linked respiration, and spare respiratory capacity (Figure 3A,C). Maximal respiration was increased by trimethoprim, but was unaltered during all other treatments. As estimated from the glycolytic proton efflux rate (PER) glycolysis was also unaltered (Figure 3B,D).

### 3.4 Effect of Inhibitors of de Novo Purine Synthesis on Insulin Signaling and Insulin‐Stimulated Glucose Uptake in L6 Myotubes

**FIGURE 4:**
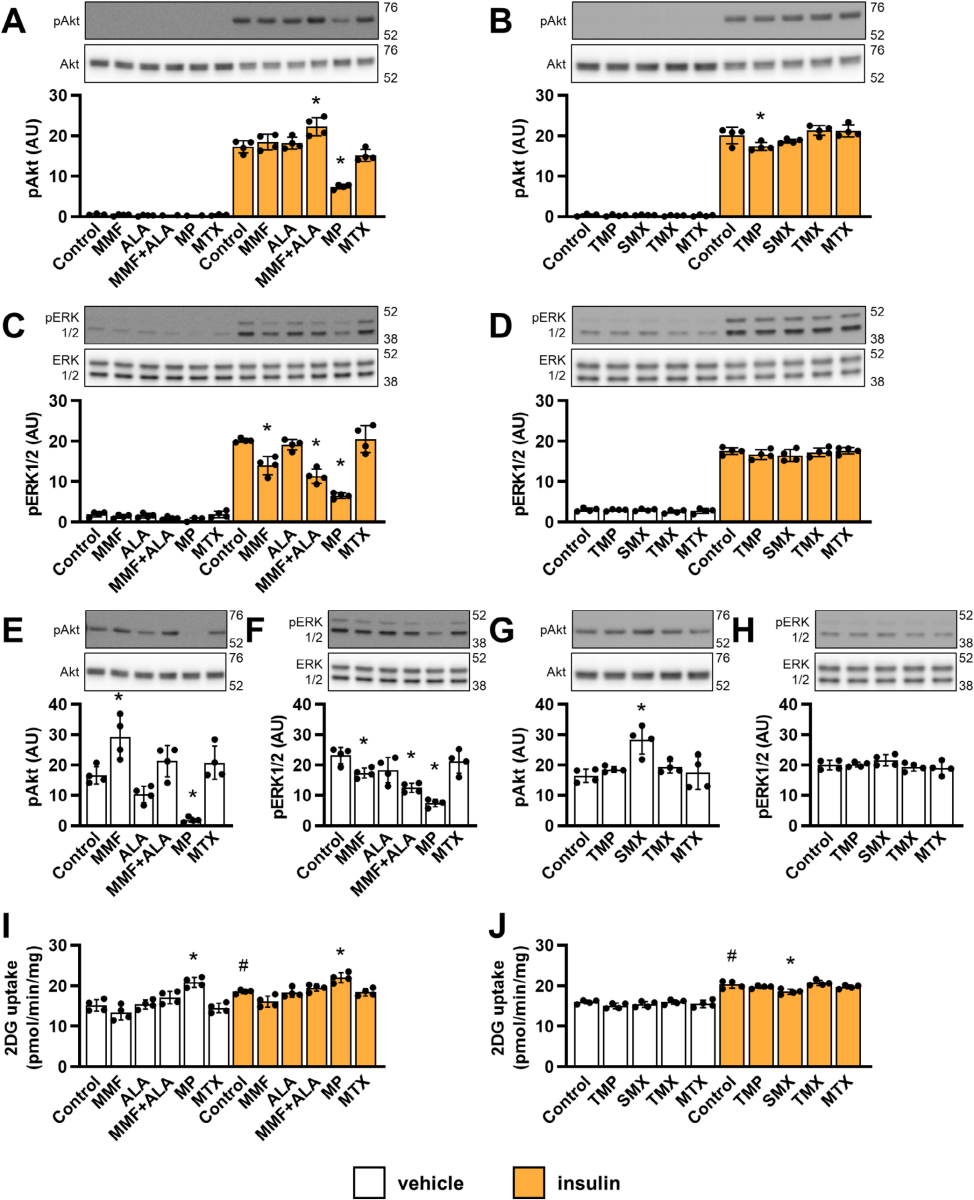
Effect of inhibitors of de novo purine synthesis on insulin signaling and insulin‐stimulated glucose uptake in L6 myotubes. L6 myotubes were incubated in nucleoside‐ and serum‐free MEMα for 24 h and treated with 5 μM mycophenolate mofetil (MMF), 5 μM alanosine (ALA), MMF + ALA, 50 μM mercaptopurine (MP), 20 μM trimethoprim (TMP), 500 μM sulfamethoxazole (SMX), 5 μM trimetrexate (TMX), 5 μM methotrexate (MTX) or vehicle (Control) for the last 16 h of these 24 h and with 120 nM insulin or vehicle for the last 20 min of these 24 h for analysis of protein phosphorylation or for the last 60 min of these 24 h for analysis of glucose uptake. Following the treatment, cells were analyzed for phospho Akt Ser473 (pAkt) and Akt (A, B, E, G) and phospho ERK1/2 Thr202/Tyr204 (pERK1/2) and ERK1/2 (C, D, F, H) with immunoblotting or for glucose uptake (I, J) with 2‐deoxy‐glucose (2DG) uptake assay. For analysis of phospho Akt and phospho ERK1/2, we recorded one short‐exposure and one long‐exposure image. The short‐exposure image was used for analysis of insulin‐induced phosphorylation (A–D), while the long‐exposure image was used for analysis of basal phosphorylation (E–H). 2DG uptake was expressed in pmol of 2DG/min/mg of total proteins. Graphs show means with SD (*n* = 4). Images show representative blots. Numbers next to blots indicate molecular weight (in kDa) of the first marker below and above the bands on the blots. (A–H) *p < 0.05 versus respective (vehicle or insulin) control, one‐way ANOVA with Dunnett's test. (I, J) *p < 0.05 versus respective (vehicle or insulin) control, # *p* < 0.05 insulin control versus vehicle control; one‐way ANOVA with Bonferroni's test. AU, arbitrary units.

Immunosuppressants and/or antineoplastics such as inhibitors of mammalian target of rapamycin (mTOR), calcineurin inhibitors, and glucocorticoids promote insulin resistance, worsen glycaemia, and/or increase the risk of diabetes [17, 18, 44, 45]. We therefore examined whether inhibitors of de novo purine synthesis might adversely affect insulin‐stimulated phosphorylation of Akt (at Ser473), phosphorylation of extracellular signal‐regulated protein kinase (ERK)1/2 (at Thr202/Tyr204), and glucose uptake (Figure 4). Mycophenolate mofetil increased basal phosphorylation of Akt (Figure 4E) while decreasing basal and insulin‐induced phosphorylation of ERK1/2 (Figure 4C,F). Alanosine tended to decrease basal phosphorylation of Akt (Figure 4E) and ERK1/2 (Figure 4F) while having no effect on insulin‐induced phosphorylation of Akt (Figure 4A) and ERK1/2 (Figure 4C). Co‐treatment with mycophenolate mofetil and alanosine increased insulin‐induced phosphorylation of Akt (Figure 4A) and decreased basal (Figure 4F) and insulin‐induced phosphorylation of ERK1/2 (Figure 4C). 6‐Mercaptopurine decreased basal and insulin‐induced phosphorylation of Akt (Figure 4A,E) and ERK1/2 (Figure 4C,F). Sulfamethoxazole increased basal phosphorylation of Akt (Figure 4G). Trimethoprim decreased insulin‐induced phosphorylation of Akt (Figure 4B). Trimetrexate (Figure 4B,D,G,H) and methotrexate (Figure 4A–H) had no effect on phosphorylation of Akt or ERK1/2. Except for 6‐mercaptopurine, which increased glucose uptake in the absence or presence of insulin (Figure 4I), and sulfamethoxazole, which reduced insulin‐stimulated glucose uptake (Figure 4J), none of the tested compounds had a significant effect on glucose transport.

### 3.5 Effect of Allopurinol on AMPK, Insulin Signaling, and Glucose Uptake in L6 Myotubes

**FIGURE 5:**
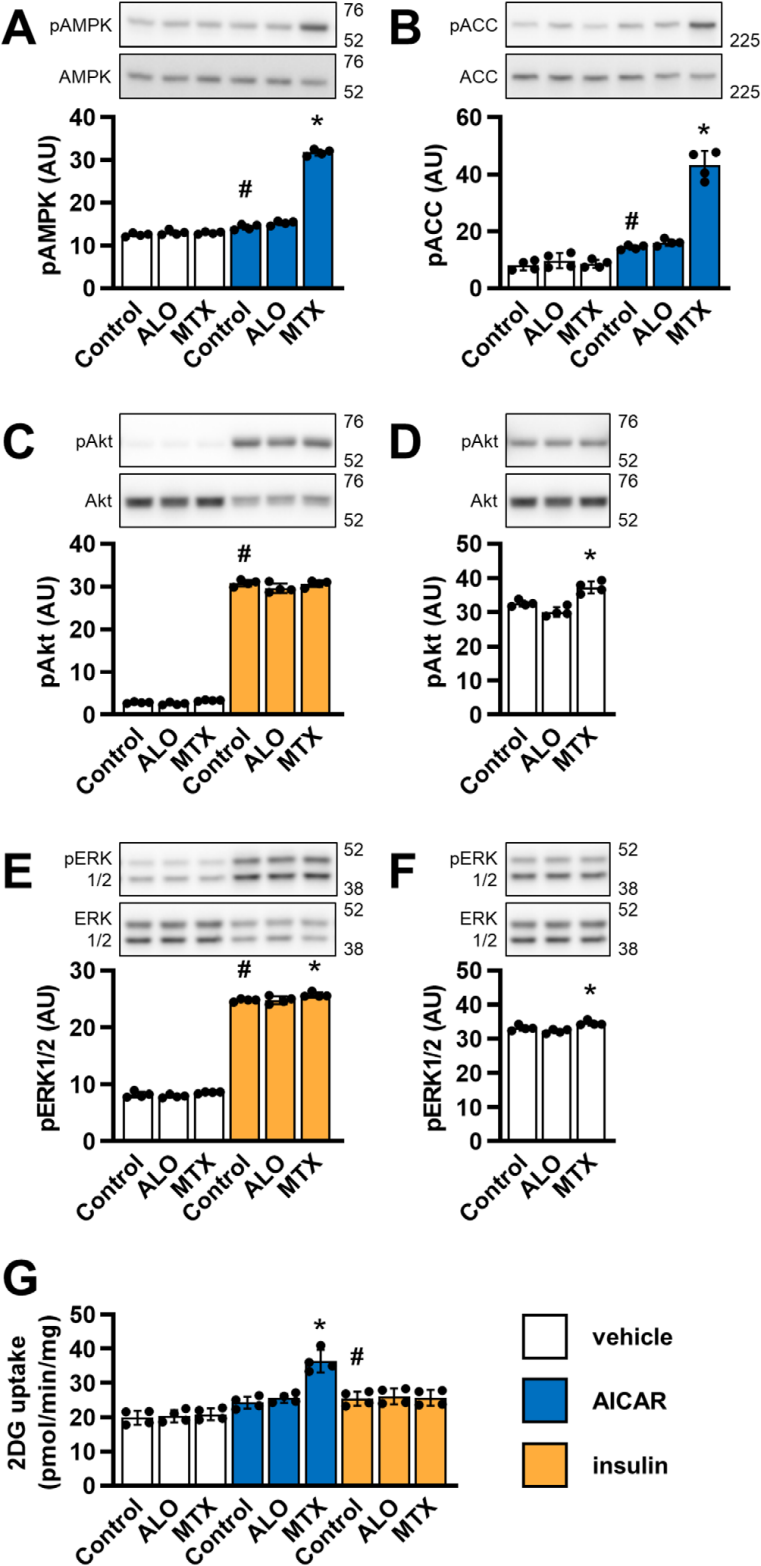
Effect of allopurinol on AMPK, insulin signaling, and glucose uptake in L6 myotubes. L6 myotubes were incubated in nucleoside‐ and serum‐free MEMα for 24 h and treated with 100 μM allopurinol (ALO), 5 μM methotrexate (MTX) or vehicle (Control) for the last 16 h of these 24 h and with 1 mM AICAR or vehicle for the last 60 min of these 24 h or with 120 nM insulin or vehicle for the last 20 min of these 24 h for analysis of protein phosphorylation (A‐F) or with 2 mM AICAR or vehicle for the last 5 h of these 24 h or with 120 nM insulin or vehicle for the last 60 min of these 24 h for analysis of glucose uptake (G). Following the treatment, cells were analyzed for phospho AMPKα Thr172 (pAMPK) and AMPKα (AMPK) (A), phospho ACC Ser79 (pACC) and ACC (B), phospho Akt Ser473 (pAkt) and Akt (C, D) and phospho ERK1/2 Thr202/Tyr204 (pERK1/2) and ERK1/2 (E, F) with immunoblotting or for glucose uptake (G) with a 2‐deoxy‐glucose (2DG) uptake assay. For the analysis of phospho Akt and phospho ERK1/2, we recorded one short‐exposure and one long‐exposure image. The short‐exposure image was used for the analysis of insulin‐induced phosphorylation (C, E), while the long‐exposure image was used for the analysis of basal phosphorylation (D, F). 2DG uptake was expressed in pmol of 2DG/min/mg of total proteins. Graphs show means with SD (*n* = 4). Images show representative blots. Numbers next to blots indicate molecular weight (in kDa) of the first marker below and/or above the bands on the blots. *p < 0.05 versus respective (vehicle, AICAR or insulin) control, #*p* < 0.05 AICAR or insulin control versus vehicle control; one‐way ANOVA with Bonferroni's test. AU, arbitrary units.

Treatment with AICAR in vivo increases purine degradation and production of uric acid, leading to hyperuricemia, which can be prevented by inhibiting xanthine dehydrogenase with allopurinol [46, 47, 48, 49] (Figure 1A). Since L6 myotubes expressed *Xdh* (Figure 1B), we asked whether allopurinol might increase AICAR action by suppressing its degradation to uric acid. In contrast to methotrexate, which augmented the effects of AICAR, allopurinol had no effect on AICAR‐induced AMPK and ACC phosphorylation and glucose uptake (Figure 5). Allopurinol also had no significant effect on insulin action.

## 4 Discussion

In this study, we found that mycophenolate mofetil (inhibitor of IMPDH), alanosine (inhibitor of ADSS), and trimetrexate (inhibitor of DHFR) promote AICAR‐induced activation of AMPK and glucose uptake in L6 myotubes. Sulfamethoxazole, an inhibitor of dihydrofolate synthesis in bacteria, also enhanced AICAR actions. These results extend our previous findings that treatment with methotrexate, an inhibitor of ATIC, or gene silencing of ATIC suppress the conversion of ZMP to IMP, thereby enhancing AICAR‐induced activation of AMPK in L6 and primary human myotubes [5, 11]. Our new results suggest that inhibition of IMPDH and ADSS, which catalyze the next two steps in de novo synthesis of GMP and AMP from IMP, or inhibition of DHFR, which maintains the pool of reduced folates required for the conversion of ZMP to IMP by ATIC, also enhance AICAR‐induced AMPK activation in L6 myotubes.

Trimetrexate, mycophenolate mofetil, and alanosine most likely enhanced AICAR‐stimulated AMPK activation by suppressing the clearance of ZMP. Once inside the cell, AICAR is phosphorylated to ZMP, which is then converted to IMP by ATIC and, subsequently, to GMP or AMP (Figure 1A). Although we did not measure intracellular nucleotides, we suspect that trimetrexate suppressed the conversion of ZMP to IMP, while mycophenolate mofetil and alanosine blocked the conversion of IMP to GMP or AMP, respectively. Consistent with this idea, trimetrexate, an inhibitor of DHFR, disrupted folate metabolism in cancer cells, which led to inhibition of ATIC and accumulation of ZMP [30]. ZMP accumulated in cancer cells also during treatment with mycophenolic acid, an active metabolite of mycophenolate mofetil [35].

Mycophenolate mofetil, alanosine, trimetrexate, and methotrexate stimulated AMPK and increased glucose uptake in the presence of AICAR, but not when they were used alone. ZMP is below the level of detection in L6 myotubes under basal conditions [5], indicating that its net rate of de novo synthesis might be so low that ZMP concentrations do not reach the threshold for AMPK activation despite inhibition of IMPDH, ADSS, or ATIC. Indeed, gene silencing of ATIC measurably increased ZMP content in L6 myotubes only when exogenous AICAR was added [5]. Differences in the rate of de novo purine synthesis perhaps explain why methotrexate is able to activate AMPK in some cell types even without the addition of exogenous AICAR [5, 50, 51, 52].

However, it also needs to be considered that drugs that we used affect more than one enzyme of the de novo purine synthesis pathway (Figure 1A). Moreover, these enzymes are not equally sensitive even to a single inhibitor such as methotrexate [3], which means that the ultimate effect of treatment with inhibitors of purine synthesis is dependent both on their concentration and duration of treatment [53]. For instance, while methotrexate or trimetrexate can produce an accumulation of ZMP due to inhibition of ATIC [30, 53], ZMP accumulation is less pronounced or does not occur with high concentrations of methotrexate or prolonged treatments [53], most likely due to inhibition of GART (Figure 1A). GART, which is required for de novo synthesis of endogenous ZMP (Figure 1A) is less sensitive to methotrexate than ATIC [3], which explains why treatment with methotrexate may block its ZMP synthesis or produce marked ZMP accumulation.

Sulfamethoxazole, a sulfonamide, also enhanced AICAR‐induced AMPK activation and glucose uptake in L6 myotubes. Interestingly, sulfamethoxazole was noted to activate AMPK in grass carp *Ctenopharyngodon idella* [54], although no mechanism of activation was established. Pharmacological compounds can activate AMPK directly by binding to it or indirectly by inducing energy stress, suppressing AMP and/or ZMP metabolism, or increasing intracellular Ca^2+^ concentrations [26, 55, 56]. However, when used alone, sulfamethoxazole did not increase phosphorylation of AMPK or ACC in L6 myotubes, indicating it acts primarily by enhancing the effects of AICAR. Sulfonamides, such as sulfamethoxazole, inhibit bacterial dihydropteroate synthase, which blocks folate synthesis, leading to inhibition of ATIC and ZMP accumulation [40]. As dihydropteroate synthase is not present in humans, this mechanism cannot explain how sulfamethoxazole enhanced the effects of AICAR. However, the anti‐rheumatic drug sulfasalazine, which contains a sulphonamide moiety, directly inhibits ATIC [57], which suggests that sulfamethoxazole might promote AICAR‐induced AMPK activation by suppressing ZMP clearance.

6‐mercaptopurine activated AMPK and increased glucose uptake in L6 myotubes in the absence of AICAR, but did not enhance AICAR action. Once inside the cell, 6‐mercaptopurine is converted to 6‐thioinosine 5′‐phosphate (aka 6‐mercaptopurine riboside 5′‐monophosphate), an AMP analogue that cannot stimulate AMPK [27] but inhibits the conversion of IMP to GMP and AMP. 6‐mercaptopurine could therefore stimulate AMPK indirectly by producing accumulation of ZMP or energy stress [58, 59, 60]. On the one hand, the mechanism involving ZMP seems less likely, since 6‐mercaptopurine did not enhance AICAR‐induced AMPK activation and glucose uptake. Indeed, co‐treatment with mycophenolate mofetil and alanosine, which would also inhibit the conversion of IMP to AMP and of IMP to GMP simultaneously, had a more potent effect on AICAR‐induced AMPK activation than mycophenolate mofetil or alanosine alone.

On the other hand, 6‐mercaptopurine could have increased endogenous ZMP without enhancing AICAR actions. Since 6‐mercaptopurine and AICAR enter cells through the same nucleoside transporter [61, 62], 6‐mercaptopurine might have reduced the uptake of AICAR, thus blocking formation of ZMP and its effects on AMPK and glucose uptake. This would be consistent with our previous findings that the presence of alternative substrates for nucleoside transporters suppresses or even abolishes response to AICAR in L6 myotubes [11]. Once inside the cell, AICAR is converted to ZMP by adenosine kinase (Figure 1A), which is inhibited by 6‐methylmercaptopurine‐riboside, a 6‐mercaptopurine metabolite [63]. Moreover, 6‐mercaptopurine blocks formation of ZMP from the corresponding base 5‐aminoimidazole‐4‐carboxamide (AICA) by inhibiting hypoxanthine phosphoribosyl transferase [64]. Clearly, 6‐mercaptopurine may have antagonized effects of AICAR by reducing its uptake and/or formation of ZMP.

6‐Mercaptopurine decreased basal and insulin‐induced phosphorylation of Akt and ERK1/2, but stimulated glucose uptake more potently than insulin, which indicates that 6‐mercaptopurine increases uptake of glucose in an insulin‐ and Akt‐independent manner. Conversely, mycophenolate mofetil and sulfamethoxazole both stimulated Akt phosphorylation without increasing glucose uptake. However, importantly, Akt phosphorylation levels in the presence of mycophenolate mofetil or sulfamethoxazole were orders of magnitude lower than those in the presence of insulin, which suggests that activation of Akt remained below the threshold for stimulation of glucose uptake. Despite the complexity of the signaling data, which will require further investigation, we can draw three general conclusions. First, drug‐induced Akt and/or ERK1/2 phosphorylation is not always associated with increased glucose uptake. Second, drug‐stimulated glucose uptake may increase even when phosphorylation of Akt and/or ERK1/2 are concurrently suppressed. Third, alterations in Akt and ERK1/2 phosphorylation in L6 myotubes are not a reliable proxy for alterations in glucose uptake.

In our study, the examined inhibitors had no significant effect on basal mitochondrial respiration and basal glycolysis in L6 myotubes, indicating they did not enhance AMPK activation and glucose uptake due to suppression of energy metabolism, which is otherwise a common characteristic of a range of AMPK activators [26]. None of the inhibitors that we tested, except for 6‐mercaptopurine, activated AMPK in the absence of AICAR, which supports the idea that energy homeostasis in L6 myotubes was maintained. However, effects on mitochondria likely depend on experimental conditions and cell type, as mycophenolate mofetil [65], 6‐mercaptopurine [66], and methotrexate [67, 68] were all shown before to affect mitochondrial membrane potential and/or oxygen consumption. Methotrexate was also previously shown to inhibit tricarboxylic acid cycle enzymes and mitochondrial respiratory chain complexes [69, 70]. Clearly, although our results suggest a mechanism whereby mycophenolate mofetil, alanosine, trimetrexate, and sulfamethoxazole promoted AMPK activation and glucose uptake in L6 myotubes via ZMP, additional suppressive effects on mitochondria under different conditions cannot be excluded.

Trimethoprim and allopurinol had no effect on AICAR‐induced AMPK activation. Trimethoprim is primarily an inhibitor of bacterial ATIC and is only a very weak inhibitor of human DHFR [43]. As trimetrexate, a potent inhibitor of human DHFR, enhanced AICAR actions, the degree of DHFR inhibition during trimethoprim treatment was likely insufficient to produce a similar effect. Unlike trimetrexate, trimethoprim did not exert almost any effect on the proliferation of myoblasts even when used in high concentrations (100 μM), which again supports the idea that DHFR remained active. On the other hand, the lack of effect of allopurinol on AMPK might be due to the fact that it acts on purine metabolism relatively far from ZMP, probably too far to lead to accumulation of ZMP. This would be consistent with the observation that allopurinol did not increase ZTP (a phosphorylated metabolite of ZMP) content in erythrocytes of patients who received allopurinol for the treatment of hyperuricaemia [21].

This study has several limitations. While we were able to establish that mycophenolate mofetil, alanosine, trimetrexate, and sulfamethoxazole promote AICAR‐induced AMPK activation and glucose uptake in L6 myotubes, we did not establish the underlying mechanisms of action. Indeed, while it seems plausible that these agents promoted AMPK activation by increasing intracellular ZMP concentrations, this is only a speculation since ZMP was not measured. Second, we did not use in vitro models of insulin resistance, which could provide additional insights concerning the effect of these agents on insulin action. Third, we can make only speculative translational inferences regarding the therapeutic or adverse effects of these agents because all our data was obtained in vitro, especially if we consider that drugs such as methotrexate can act as AMPK activators or as enhancers of AICAR‐induced AMPK activation depending on the cell type and/or conditions in the cell [5]. For instance, while we can speculate that mycophenolate mofetil could provide protection against diabetes by promoting AMPK activation via ZMP in patients with autoimmune diseases or transplant recipients [16, 17, 18], in vivo approaches will be needed to test this assertion. Similarly, while AMPK activation via ZMP might potentially contribute to the hypoglycaemic effects of sulfamethoxazole [71], in vivo experiments will be needed to examine this question.

In summary, our results show that mycophenolate mofetil, alanosine, trimetrexate, and sulfamethoxazole mimic methotrexate's effects by enhancing AICAR‐induced AMPK activation and glucose uptake in L6 myotubes. Collectively, our results indicate that IMP metabolism serves as a gateway for the modulation of AMPK and its metabolic effects in skeletal muscle cells. Furthermore, they suggest that purine synthesis inhibitors may help protect against metabolic dysregulation via AMPK activation.

## Conflicts of Interest

The authors declare no conflicts of interest.

## Supporting information


**Data S1:** Supporting Information.

## Data Availability

The data that supports the findings of this study are available in the Supporting Information of this article.

## Endnotes

^1^
10‐CHO‐THF, N^10^‐formyl‐THF; 2DG, 2‐deoxy‐glucose; 5,10‐CH_2_‐THF, N^5^,N^10^‐methylene THF; ACC, acetyl‐coenzyme A carboxylase; Actb, actin beta; ADSL, adenylosuccinate lyase; ADSS, adenylosuccinate synthetase; AICAR, 5‐aminoimidazole‐4‐carboxamide ribofuranoside; ALA, alanosine; ALO, allopurinol; AMP, adenosine monophosphate; AMPK, AMP‐activated protein kinase; ATIC, 5‐aminoimidazole‐4‐carboxamide ribonucleotide formyltransferase/inosine monophosphate cyclohydrolase; ATP, adenosine triphosphate; DHF, dihydrofolate; DHFR, dihydrofolate reductase; dTMP, deoxythymidine monophosphate; dUMP, deoxyuridine monophosphate; EC50, half‐maximal effective concentration; ECAR, extracellular acidification rate; ECL, enhanced chemiluminescence; ERK, extracellular signal‐regulated protein kinase; FAO, fatty acid oxidation; FBS, fetal bovine serum; FCCP, carbonyl cyanide 4‐(trifluoromethoxy)phenylhydrazone; FGAR, formylglycinamide ribonucleotide; GAR, glycinamide ribonucleotide; GART, glycinamide ribonucleotide formyltransferase; GMP, guanosine monophosphate; GMPS, guanosine monophosphate synthetase; GPAT, glutamine phosphoribosylpyrophosphate amidotransferase; Hx, hypoxanthine; IMP, inosine monophosphate; IMPDH, inosine monophosphate dehydrogenase; mAb, monoclonal antibody; MMF, mycophenolate mofetil; MP, mercaptopurine; MTX, methotrexate; OCR, oxygen consumption rate; pAb, polyclonal antibodies; PBS, phosphate‐buffered saline; PER, proton efflux rate; PRA, phosphoribosylamine; PRPP, 5‐phosphoribosyl‐1‐pyrophosphate; PVDF, polyvinylidene fluoride; qPCR, quantitative real‐time polymerase chain reaction; SAICAR, N‐succinyl‐5‐aminoimidazole‐4‐carboxamide ribonucleotide; SD, standard deviation; SDS, sodium dodecyl sulfate; SMX, sulfamethoxazole; TBST, tris‐buffered saline with Tween 20; THF, tetrahydrofolate; TMP, trimethoprim; TMX, trimetrexate; TS and Tyms, thymidylate synthetase (protein and gene, respectively); v/v, volume per volume; w/v, weight per volume; Xan, xanthine; XDH, xanthine oxidase; XMP, xanthosine monophosphate; ZMP, 5‐aminoimidazole‐4‐carboxamide ribonucleotide.
