# Supplementary material for: Diverse Inhibitors of De Novo Purine Synthesis Promote AICAR‐Induced AMPK Activation and Glucose Uptake in L6 Myotubes
Source: Biofactors. 2025 Aug 12;51(4):e70037. doi: 10.1002/biof.70037 (PMC12341450; doi:10.1002/biof.70037)

Diverse inhibitors of de novo purine synthesis promote AICAR-induced AMPK activation and glucose uptake in L6 myotubes

Klemen Dolinar[a], Katarina Miš[a], Katja Šopar[a], Mateja Šutar[a], Meta Božič[a], Matic Kolar[a], Tim Hropot[a], Pablo M Garcia-Roves[b,c], Alexander V Chibalin[d,e], Sergej Pirkmajer[a,*]

[a]Institute of Pathophysiology, Faculty of Medicine, University of Ljubljana, Ljubljana, Slovenia
[b]Department of Physiological Sciences, Faculty of Medicine and Health Sciences, University of Barcelona, Barcelona, Spain
[c]Nutrition, Metabolism and Gene Therapy Group, Diabetes and Metabolism Program, Institut d'Investigació Biomédica de Bellvitge (IDIBELL), Barcelona, Spain
[d]Department of Molecular Medicine and Surgery, Integrative Physiology, Karolinska Institutet, Stockholm, Sweden
[e]National Research Tomsk State University, Tomsk, Russia

MEMBRANES and BLOTS

Membranes and blots for Figure 1

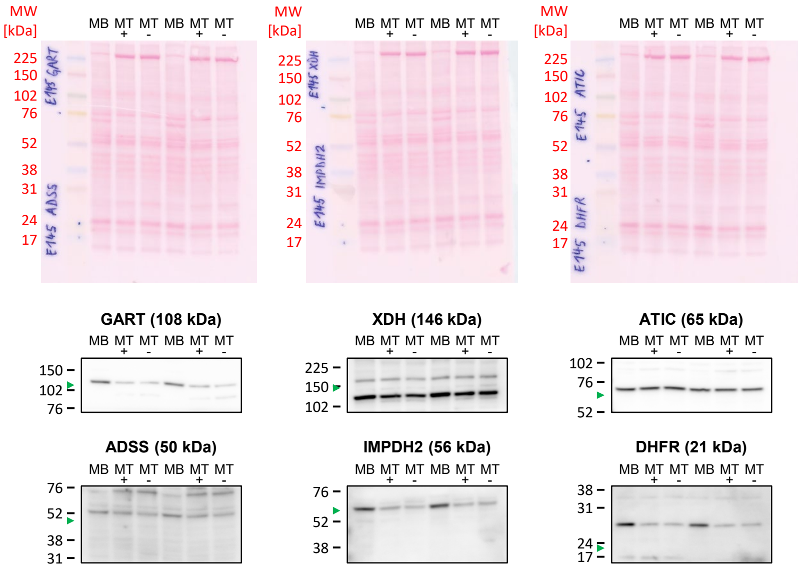

Membranes and blots for Figure 2 (1/3)

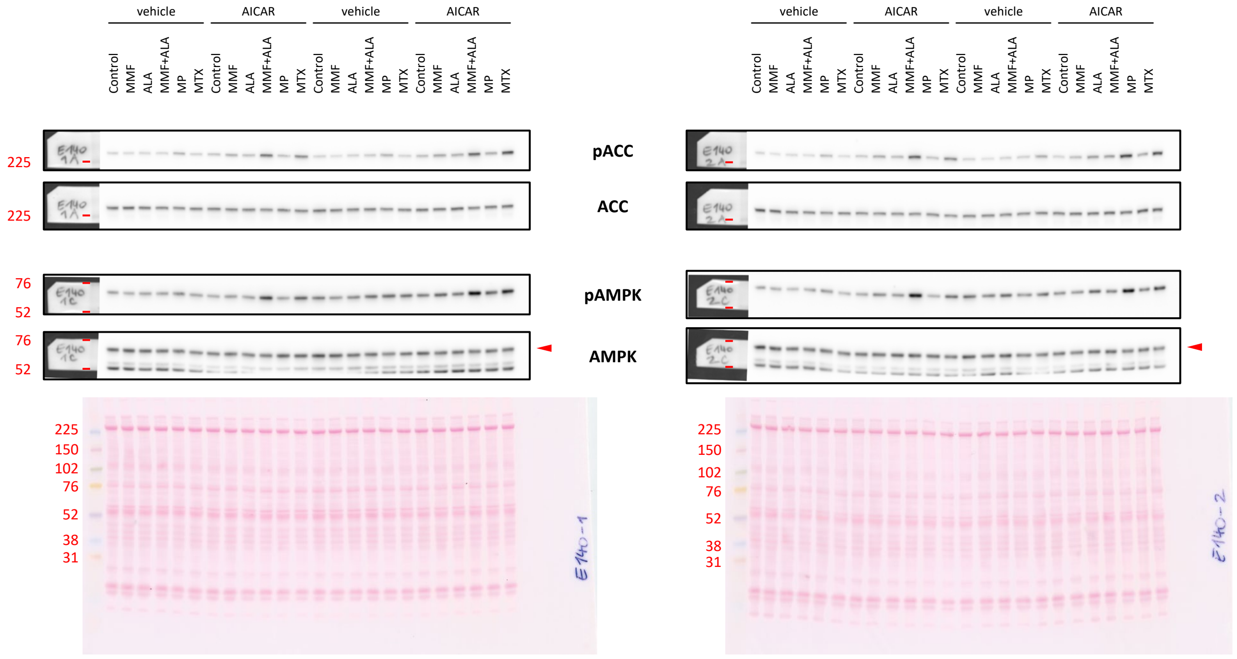

Membranes and blots for Figure 2 (2/3)

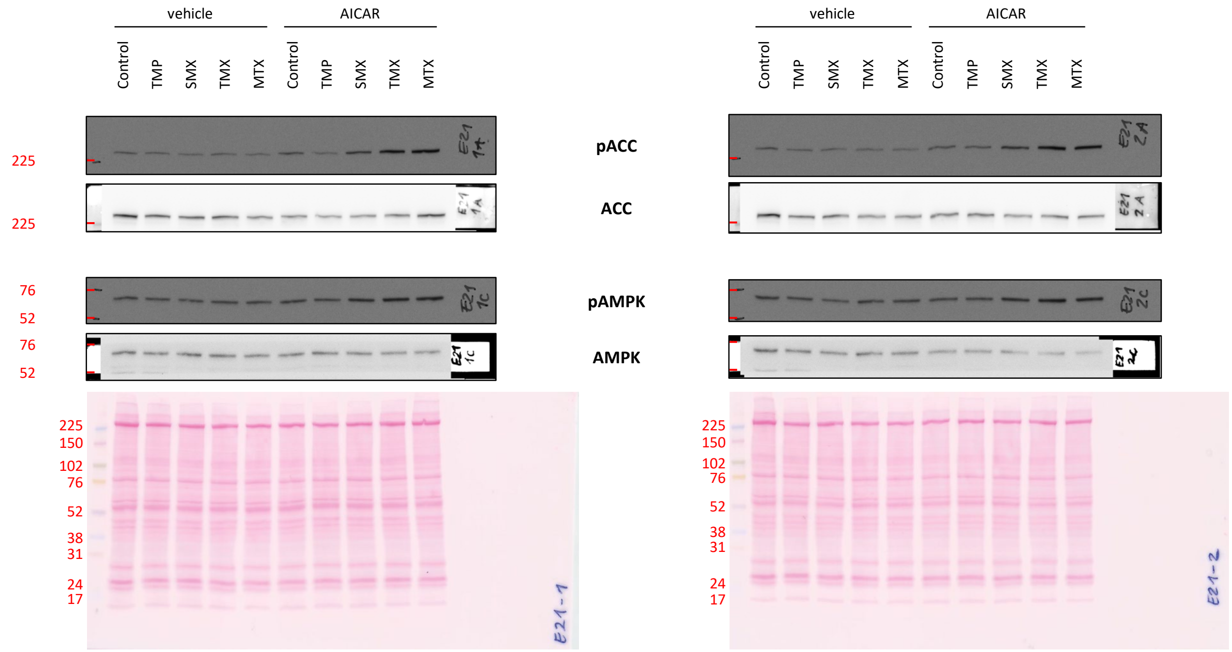

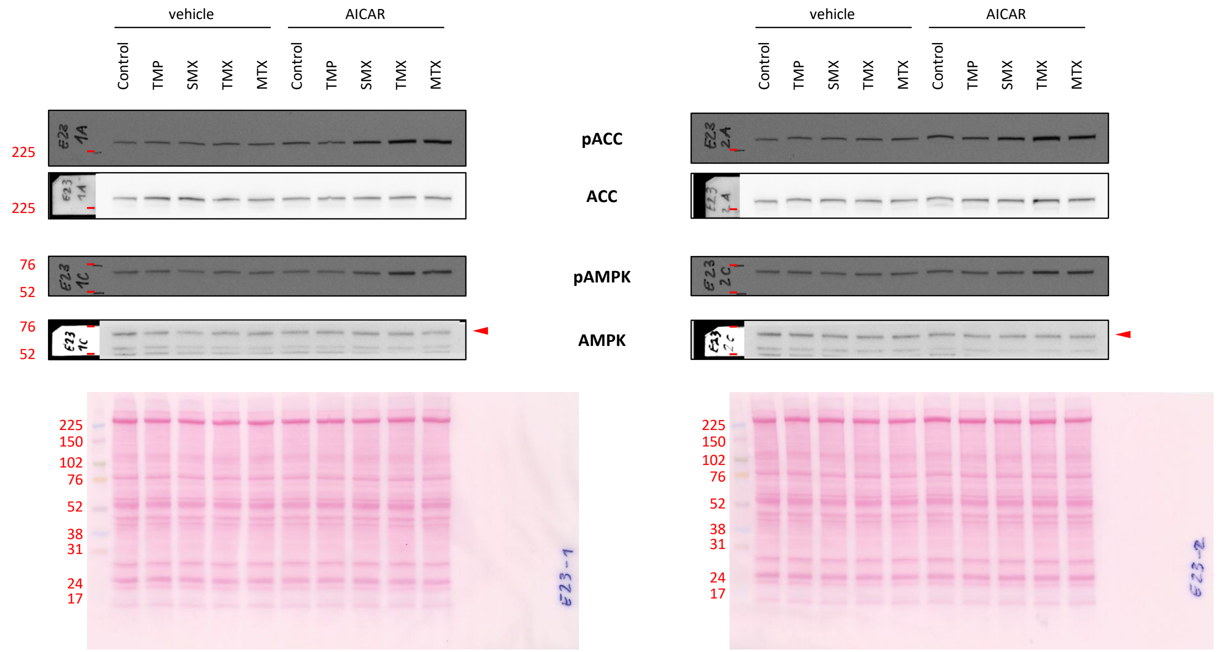

Membranes and blots for Figure 2 (3/3)

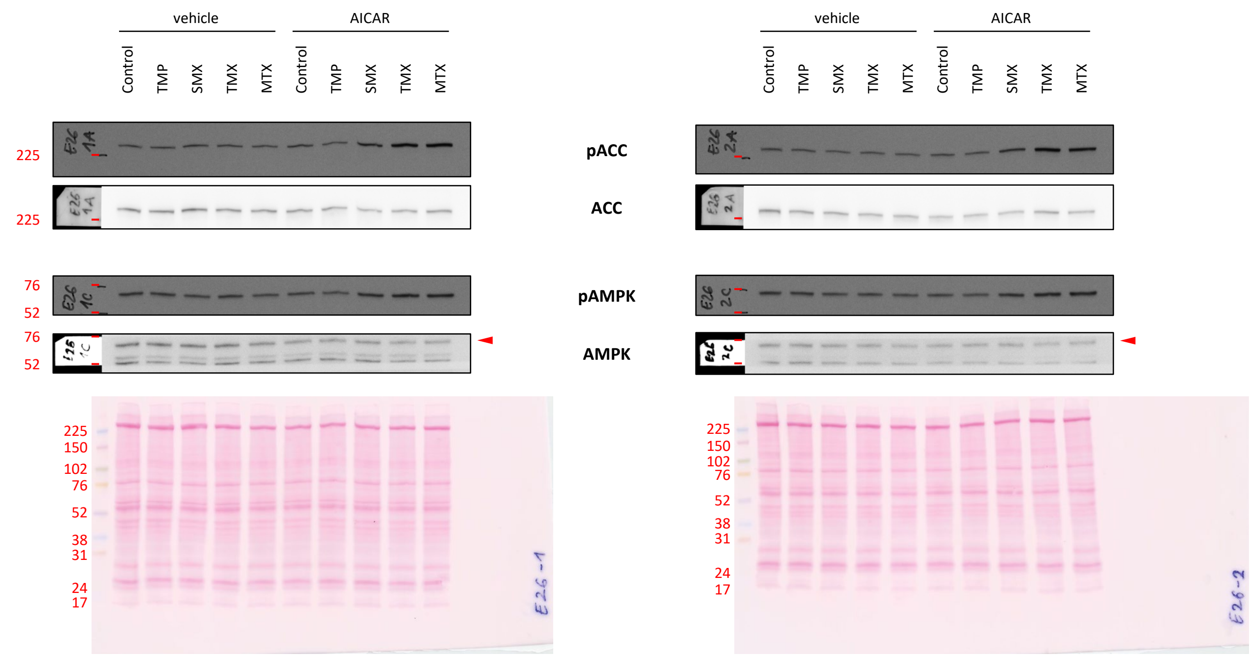

Membranes and blots for Figure 4

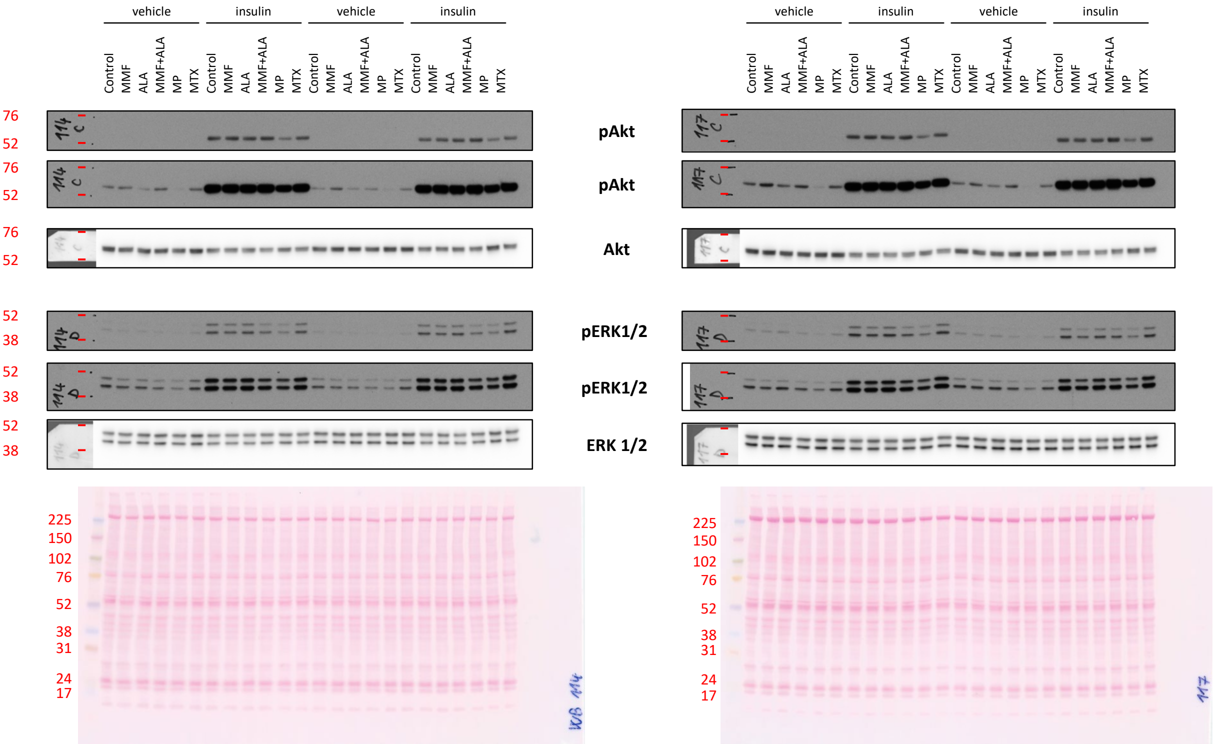

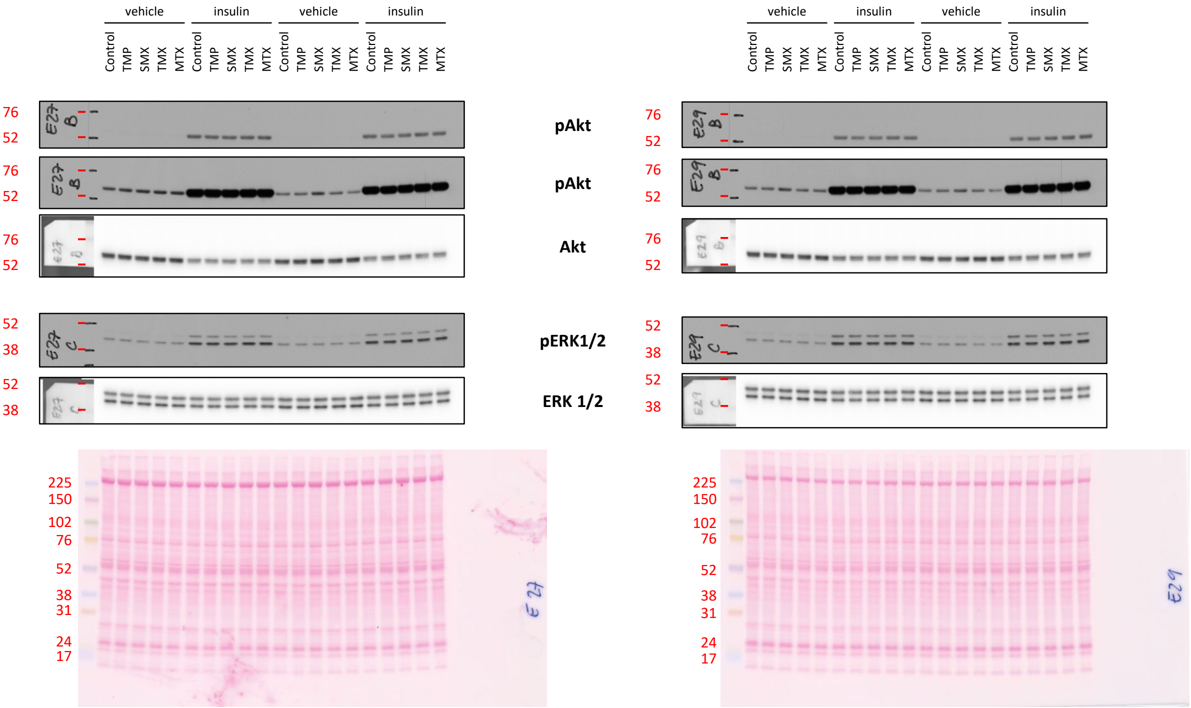

Membranes and blots for Figure 5

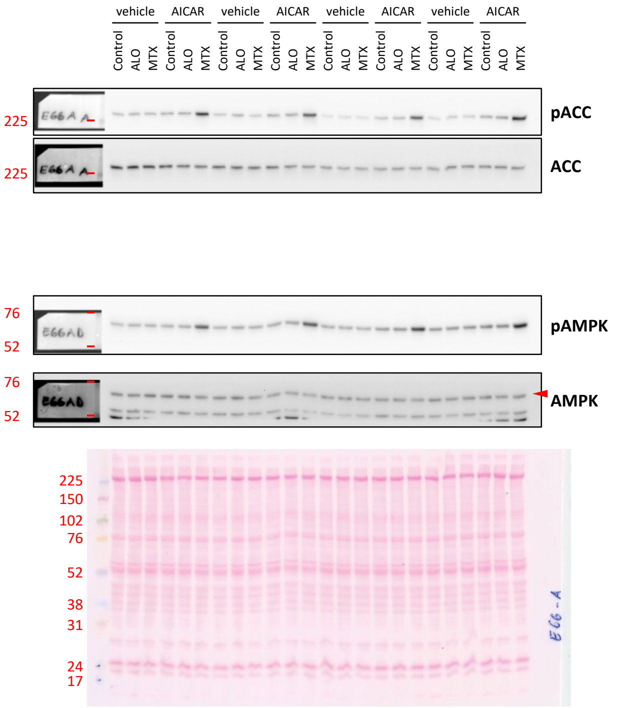

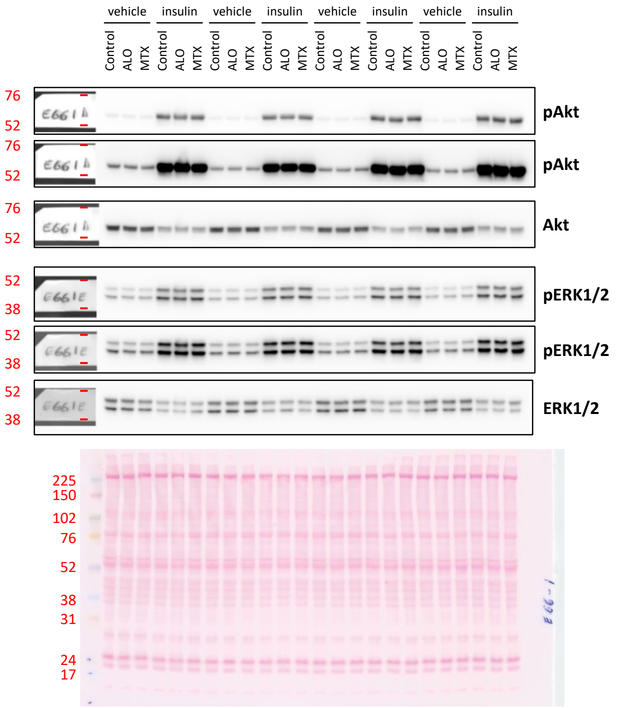

Supplement: Supplementary file 1 — Data S1: Supporting Information. [file BIOF-51-0-s001.zip › biof70037-sup-0001-supporting_information.pdf]
